# Simplified scoring of the Actionable 8-item screening questionnaire for neurogenic bladder overactivity in multiple sclerosis: a comparative analysis of test performance at different cut-off points

**DOI:** 10.1186/s12894-015-0100-z

**Published:** 2015-10-24

**Authors:** Peter Joseph Jongen, Bertil F. Blok, John P. Heesakkers, Marco Heerings, Wim A. Lemmens, Rogier Donders

**Affiliations:** Department of Community and Occupational Medicine, University Groningen, University Medical Centre Groningen, Antonius Deusinglaan 1, 9713 AV Groningen, The Netherlands; MS4 Research Institute, Ubbergseweg 34, 6522 KJ Nijmegen, The Netherlands; Department of Urology, Erasmus Medical Center, P.O. Box 2040, 3000 CA Rotterdam, The Netherlands; Department of Urology, Radboud University Nijmegen Medical Centre, P.O. Box 9101, 6500 HB Nijmegen, The Netherlands; MH-advies & organisatiebureau, IJselstraat 81, 9406 TR Assen, The Netherlands; National MS Foundation The Netherlands, Mathenesserlaan 378, 3023 HB Rotterdam, The Netherlands; Department for Health Evidence, Radboud University Nijmegen Medical Centre, P.O. Box 9101, 6500 HB Nijmegen, The Netherlands

## Abstract

**Background:**

The Actionable questionnaire is an 8-item tool to screen patients with multiple sclerosis (MS) for neurogenic bladder problems, identifying those patients who might benefit from urological referral and bladder-specific treatment. The original scoring yields a total score of 0 to 24 with cut-off point 6. A simplified scoring, yielding a total score of 0 to 8 with cut-off point 3, has been developed in urogynaecological patients, but has not been investigated in MS.

**Methods:**

One-hundred-and-forty-one MS patients completed the Actionable on two occasions. We compared the test performance of the simplified scoring with cut-off point 3 with that of cut-off point 2, using the original scoring with cut-off point 6 as a gold standard. The following measures were calculated: True Positives (TP), True Negatives (TN), False Positives (FP), False Negatives (FN), Sensitivity, Specificity, Positive Predictive Value (PPV), Negative Predictive Value (NPV), and Accuracy. The associations between positive test result and urological treatment, and bladder-specific drug treatment were calculated.

**Results:**

For cut-off point 3 the outcomes (Test 1, Test 2) were: TP 43.26 %, 40.88 %; TN 29.79 %, 32.85 %; FP 0.00 %, 0.00 %; FN 26.95 %, 26.28 %; Sensitivity 0.62, 0.61; Specificity 1.00, 1.00; PPV 1.00, 1.00; NPV 0.53, 0.55; Accuracy 0.73, 0.74; and for cut-off point 2: TP 59.57 %, 59.85 %; TN 26.95 %, 31.39 %; FP 2.84 %, 1.46 %; FN 10.63 %, 7.30 %; Sensitivity 0.85, 0.89; Specificity 0.90, 0.96; PPV 0.95, 0.98; NPV 0.72, 0.81; Accuracy 0.87, 0.91.  Cut-off 3 completely prevented FP outcomes, but wrongly classified 26 % of the patients as negative (FN). Cut-off 2 reduced the FN to 7–10 %, with low FP values (2.84–1.46 %). With cut-off 2, the percentage of patients screened positive was higher in the Progressive group (75.00 %) than in the Relapsing Remitting group (56.25 %) (*P* = 0.0331), which was not the case with cut-off 3. Only a positive test according to the original scoring was associated with both urological treatment (*P* = 0.0119) and bladder-specific medication (*P* = 0.0328).

**Conclusions:**

Our findings suggest that in MS patients the simplified Actionable scoring is more accurate with cut-off point 2 than with cut-off point 3, especially by substantially reducing FN outcomes; and that in MS the original Actionable scoring seems preferable.

## Background

The Actionable questionnaire is a short, 8-item, psychometrically validated instrument that was developed in response to the growing need to screen patients with multiple sclerosis (MS) for neurogenic bladder problems [1]. The questionnaire accurately identifies MS patients with bladder symptoms who might benefit from urological referral and specific treatment [1]. It does so by asking questions that uncover whether patients are experiencing urinary symptoms, to what degree, and what the effects of these symptoms are on daily life [1]. The Actionable is a shortened version of the 16-item Actionable Bladder Symptom Screening Tool (ABSST), that was developed with the same purpose [2]. Both the Actionable and the ABSST have been demonstrated to have a high sensitivity and a high specificity of approximately 85 to 90 % [1, 2].

As reported by Bates et al. [1] the item scoring of the Actionable is similar to that of the ABSST: each item is scored on a 4-point scale that is indicated by boxes, ranging from no symptoms or impact (most left hand box) to extreme symptoms or impact (most right hand box). From left to right the boxes are scored as 0, 1, 2 and 3. The Actionable score is calculated as the sum of the eight item scores and ranges from 0 (minimum) to 24 (maximum). The cut-off point for positivity is 6 [1].

In a recent study Cardozo et al. validated the Actionable questionnaire also as a screening tool in women with incontinence due to an overactive bladder, demonstrating a sensitivity of 79 % and a specificity of 98 % [3]. However, in that study the item scoring was different from that described by Bates et al. [1]: any checked 3^rd^ or 4^th^ right hand box incurred a point, and consequently the Actionable score ranged from 0 (no checked 3^rd^ or 4^th^ right hand box) to 8 (8 checked 3^rd^ or 4^th^ hand boxes). It is conceivable that this simplified scoring system appeals to health care professionals using the Actionable questionnaire as a routine standard of care in their daily practice, as well as to patients who use the questionnaire for self-screening in the context of self-management. Interestingly, in the simplified scoring the cut-off point indicating a need for urological evaluation of bladder function was 3 [3], whereas intuitively a cut-off point of 6 in a scale from 0 to 24 would be expected to result in a cut-off point of 2 in a scale from 0 to 8.

In view of the established validity of the simplified Actionable scoring in women with overactive bladder symptoms, we asked ourselves whether the scoring would also be appropriate for use in patients with MS and what would be the best cut-off point. So, we compared the test performance of the cut-off points 3 and 2 in the simplified scoring (0 to 8), using the original scoring (0 to 24) with cut-off point 6 as a gold standard [1]. Here we report the results.

## Methods

### Study design and setting

The study was part of the Dutch Actionable validation study, an observational non-interventional web-based study in The Netherlands in the period January 2015 to May 2015. The Dutch Actionable validation study assessed the test-retest reliability and concurrent validity of a Dutch version of the English Actionable questionnaire. The study was investigator-initiated and investigator-sponsored with financial support from Allergan Pharmaceutical Ireland Inc. The inclusion criteria for participation were 1) having been diagnosed with MS, 2) no relapse in the last 30 days, and 3) willing and able to comply with the requirements of the protocol, i.e. online completion of questionnaires and having the Expanded Disability Status Scale (EDSS) score assessed by phone. Patients were informed by the urological departments of the Erasmus Medical Centre and the Radboud University Nijmegen Medical Centre, neurologists and MS nurses, the websites of patient organizations and of the MS4 Research Institute. The study information and the consent form were available as download on the website of the MS4 Research Institute www.ms4ri.nl. After having given their written consent patients received a personal code and logged on to the website, to choose a username and password. The study was performed using the LimeSurvey software, an open source online application. To protect the personal data from unauthorized access various mechanisms were used, among others the use of a personal username and a strong password, separation in the database of personal information from the answers to the questions, each screen having a username and password protection, Virtual Private Network (VPN) tunnelling, 256-bits encryption, and the encryption of the participants’ identities via unique 15 digits codes. Automated completeness checks were done before questionnaires could be submitted. The help desk (MH) contacted respondents by phone in case they did not succeed in completing questionnaires. The study protocol was presented to the ethical committee Medisch Ethische Toetsingscommissie Brabant (nr NW2015-08) and the committee concluded that a review was not indicated, as the study did not qualify for being tested according to the Dutch Medical Research Involving Human Subjects Act of 1999 (http://wetten.overheid.nl/BWBR0009408) [4]. The study was performed in agreement with the Declaration of Helsinki.

Disability was assessed by use of the EDSS score via telephone. The EDSS is a generally accepted and widely used measure of MS-related disability. The classical EDSS is based on a neurological examination that provides the basis for the assessment of several functional systems (visual, brainstem, pyramidal, cerebellar, sensory, bowel and bladder, cerebral) that, according to predefined algorithms, contribute to the EDSS score. An EDSS version for use by telephone via a structured interview has been developed and validated [5].

### Actionable questionnaire

**Fig. 1:**
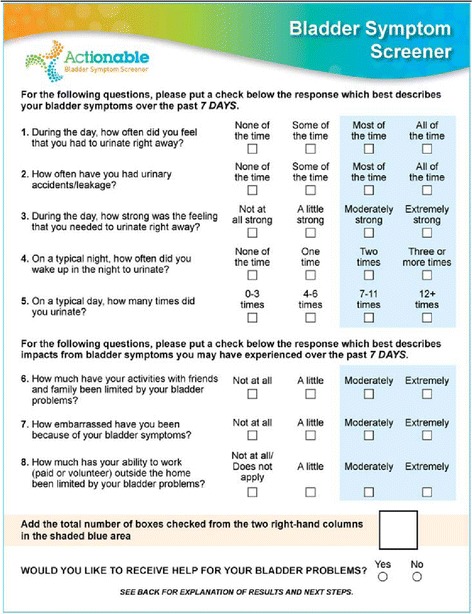
Actionable 8-item screening questionnaire for neurogenic bladder overactivity in multiple sclerosis

The Actionable questionnaire was completed at baseline (Day 1) and after 1 week (Day 8), primarily for assessment of the test-retest reliability of the Dutch version. For each of the assessments the Actionable score was calculated in two ways. Firstly, according to the original description by Bates et al., each item was scored on a 4-point scale indicated by boxes, ranging from no symptoms or impact (most left hand box) to extreme symptoms or impact (most right hand box) [1]. From left to right the boxes were scored as 0, 1, 2 and 3. The Actionable score was calculated as the sum of the eight item scores and ranged from 0 (minimum) to 24 (maximum). Secondly, according to the description by Cardozo et al., after having scored the items on a 4-point scale as mentioned above, any checked 3^rd^ or 4^th^ right hand box incurred a point [3] (Fig. 1). Thus, the simplified Actionable score ranged from 0 (no checked 3^rd^ or 4^th^ right hand box) to 8 (eight checked 3^rd^ or 4^th^ hand boxes).

### Statistics

To assess in MS patients the performance of the cut-off points 3 and 2 in the simplified Actionable scoring (0 to 8), the following measures were calculated, using the original scoring (0 to 24) with cut-off point 6 as a gold standard: the number of True Positives (TP), the number of True Negatives (TN), the number of False Positives (FP), the number of False Negatives (FN), Sensitivity TP/(TP + FN), Specificity TN/(TN + FP), Positive Predictive Value (PPV) TP/(TP + FP), Negative Predictive Value (NPV) TN/(TN + FN), and Accuracy (TP + TN)/(Positives + Negatives) [6]. Differences between groups for dichotomous variables were tested using the Chi-square test. *P* values lower than 0.05 were considered statistically significant.

## Results

One-hundred-and-forty-one patients were included in the study, 106 females and 35 males. The mean (standard deviation [SD]) age was 47.7 (10.40) years (minimum 24 years, maximum 73 years), the mean (SD) disease duration 10.1 (8.0) years (minimum 0 years, maximum 35 years), and the mean (SD) EDSS score 4.3 (1.8) (minimum 0.00, maximum 7.5). Eighty patients were relapsing remitting, 48 progressive and in 13 patients the disease course was unknown. Forty-five (31.91 %) patients were on bladder medication, whereas 96 (68.09 %) were not. Fifty-six (39.72 %) patients were seen by an urologist for overactive bladder symptoms, whereas 85 (60.28 %) were not. The Day 8 assessment was completed by 137 patients.

The mean, SD, minimum and maximum values of the Actionable scores at Day 1 (Test 1) and at Day 8 (Test 2) according to the original scoring system (0 to 24) and to the simplified scoring system (0 to 8) are presented in Table 1. The ratio between the mean score in the simplified scoring (0 to 8) and the mean score in the original scoring (0 to 24) was 0.3050 at Test 1 and 0.2972 at Test 2. Table 2 shows the numbers of patients tested positive and tested negative with the use of cut-off point 3 and with the use of cut-off point 2, as compared to the test results of the original scoring (cut-off point 6) at Test 1. Table 3 shows this information for Test 2. Table 4 presents the statistical measures for the cut-off points 3 and 2 in the simplified scoring, using the original scoring with cut-off 6 as a gold standard. At both tests the use of cut-off point 3 resulted in the complete absence of false positive patients, but also in a false negative result in 38 patients (26.95 % of the total population) at Test 1 and in 36 patients (26.27 % of the total population) at Test 2. Accordingly, the sensitivity and specificity at cut-off point 3 were 0.62, 0.61 and 1.00, 1.00 (Test1, Test 2). In contrast, at cut-off point 2 the numbers of false positive and false negative patients were 4 (2.84 %) and 15 (10.64 %) at Test 1, and 2 (1.46 %) and 10 (7.30 %) at Test 2, the sensitivity was 0.95, 0.89 and the specificity 0.90, 0.96 (Test 1, Test 2). The differences in these performance measures between the cut-off points 3 and 2 were reflected by the accuracy, being 0.73, 0.74 at cut-off 3 and 0.87, 0.91 at cut-off 2 (Test 1, Test 2).

**Table 1:** Actionable scores according to the original scoring system (0 to 24) and the simplified scoring system (0 to 8) in MS patients at two assessments with a week interval

	Patients	Mean	SD	Minimum	Maximum
Test 1 original scoring	*N* = 141	8.0000	3.9943	0	18
Test 2 original scoring	*N* = 137	7.6131	4.0041	0	21
Test 1 simplified scoring	*N* = 141	2.4397	1.8220	0	8
Test 2 simplified scoring	*N* = 137	2.2628	1.7710	0	7

**Table 2:** Numbers of patients tested positive and tested negative with the simplified scoring using the cut-off points 3 and 2, compared to the original scoring (cut-off point 6) at Test 1

Cut-off point 3 vs. original scoring				Cut-off point 2 vs. original scoring			
	> = 6	<6	Total		> = 6	<6	Total
> =3	61	0	61	> = 2	84	4	88
<3	38	42	80	<2	15	38	53
Total	99	42	141	Total	99	42	141

**Table 3:** Numbers of patients tested positive and tested negative with the simplified scoring using cut-off points 3 and 2 compared to the original scoring (cut-off point 6) at Test 2

Cut-off point 3 vs. original scoring				Cut-off point 2 vs. original scoring			
	> = 6	<6	Total		> = 6	<6	Total
> = 3	56	0	56	> = 2	82	2	84
<3	36	45	81	<2	10	43	53
Total	92	45	137	Total	92	45	137

**Table 4:** Performance measures of the simplified scoring (0 to 8) with cut-off points 3 and 2, using the original scoring system (0 to 24) and cut-off point 6 as a gold standard

Measures of performance	Test 1 (*N* = 141)		Test 2 (*N* = 137)	
	Cut-off	Cut-off	Cut-off	Cut-off
	Point 3	Point 2	Point 3	Point 2
True Positives	43.26 %	59.57 %	40.88 %	59.85 %
True Negatives	29.79 %	26.95 %	32.85 %	31.39 %
False Positives	0.00 %	2.84 %	0.00 %	1.46 %
False Negatives	26.95 %	10.63 %	26.28 %	7.30 %
Sensitivity	0.62	0.848	0.608	0.891
Specificity	1.00	0.904	1.00	0.956
Positive Predictive Value	1.00	0.954	1.00	0.976
Negative Predictive Value	0.525	0.716	0.555	0.811
Accuracy	0.730	0.865	0.737	0.912

For each of the three modes of screening (original with cut-off 6, simplified with cut-off 3, simplified with cut-off 2) the percentages of positive and negative patients that were treated by an urologist for MS-related bladder problems, and the percentages of patients that were taking medication for MS-related bladder problems are presented in Table 5. Differences between the percentages of positive and negative patients in both categories only occurred in the original scoring system.

**Table 5:** Percentages of MS patients treated by an urologist and percentages receiving bladder-specific drug treatment in relation to positive and negative test results according to the original scoring and simplified scoring systems

*N* = 141	Original scoring cut-off 6		Simplified scoring cut-off 3		Simplified scoring cut-off 2	
	Positive	Negative	Positive	Negative	Positive	Negative
	70.21 %	29.79 %	43.26 %	56.74 %	62.41 %	37.59 %
Treated by urologist	46.46 %	23.81 %*	50.82 %	31.25 %**	44.32 %	32.08 %
Bladder medication	37.37 %	19.05 %***	36.07 %	28.75 %	38.64 %	20.75 %****

*P = 0.0119; **P = 0.0186; ***P = 0.0328; ****P = 0.0274

When comparing the RRMS group with the progressive group, it was found that with the original scoring (0 to 24, cut-off 6) and with the simplified scoring and cut-off 2, the percentage of patients that was screened positive was higher in the progressive group (85.42 and 75.00 %, respectively) than in the RRMS group (65.00 and 56.25 %, respectively) (*P* = 0.0121 and *P* = 0.0331, respectively). This was not the case with the use of the simplified scoring and cut-off 3 (54.17 % vs. 38.75 %; *P* = 0.0893).

## Discussion

In contrast to the original scoring of the Actionable questionnaire [1], the simplified scoring (0 to 8) had, to our knowledge, not previously been investigated in MS patients [1–3]. We assessed the test performance of the cut-off points 3 and 2 in the simplified scoring, using the original scoring with cut-off 6 as a gold standard. It was found that the application of cut-off 3 completely prevented patients from being falsely screened as positive (specificity 1.00). However, it was also observed that about one in four patients (26 %) was wrongly classified as negative, and thus failed to be identified as a candidate for urological referral. In contrast, the use of cut-off 2 prevented a false negative qualification in about two out of three patients that were classified as such by cut-off 3, whereas a false positive test result occurred in only 4 (2.84 %) and 3 (2.19 %) patients of the total group. When considering accuracy as an overall measure of test performance, it appears that the use of cut-off point 3 achieved less than the use of cut-off 2: about 0.73 vs. 0.89.

We hypothesized that a positive test result would be associated with urological treatment and with bladder-specific medication. Only a positive test according to the original scoring with cut-off 6 was indeed associated with these two conditions, whereas the simplified scoring with cut-off 3 was associated only with urological treatment, and the simplified scoring with cut-off 2 only with the use of bladder-specific medication. This suggests that the original scoring might result in a more valid screening, which could relate to the more differentiated scoring system of 0 to 24.

The study data were acquired via patients’ self-report. Given the well-known prevalence of cognitive impairment in patients with MS [7], it may be thought that cognitive impairment may have interfered with the quality of our data, and thus with the study’s conclusions. However, Gold et al. have demonstrated that cognitive impairment in MS patients does not affect the reliability and validity of self-report health measures [8]. Therefore, we are confident that the quality of our outcomes were not affected by cognitive dysfunction.

The evaluation of a screening tool generally focuses on specificity, as this measure has a greater impact on predictive values [3]. In a study in women with incontinence due to overactive bladder, it was demonstrated that the use of the Actionable questionnaire with cut-off score 3 strongly distinguishes between patients who should be treated vs. those who do not require treatment [3]. A total score of ≥ 3 showed a sensitivity of 0.79 and a specificity of 0.98 with respect to the clinician-based assessment of whether or not treatment was needed [3]. In MS patients, however, the use of cut-off point 3 has not been validated by sensitivity and specificity values relating to clinician-based evaluations. In our study we also did not relate the scores to the outcome of clinical assessments, but by using the original scoring with cut-off 6 as a gold standard we approximated the Actionable’s performance values in MS patients. Our data suggest that in MS the use of cut-off 3 is indeed associated with a very high specificity (1.00), but unintentionally results in a possibly unacceptably high number of patients (26 %) that, although being screened negative, actually might need urological referral. Remarkably, the use of cut-off point 2 resulted in both a high specificity of 0.90–0.96, and a high sensitivity of 0.85–0.89. Yet, the comparison of the performance values obtained in our MS study with those in the urogynaecological patients is indirect, as the comparator in the latter was a clinician-based assessment [3]. It is our intention to perform a prospective study on the use and the added value of the Actionable questionnaire in real-life practice in The Netherlands, with instrumental urological assessments, such as flowmetry, post-void residual assessment or cystometry, and a comparison of the original scoring with the two simplified scorings.

Circumstantial evidence in favour of cut-off point 2 in MS, is the fact that application of the ratio of means (0.3050 at Test 1 and 0.2972 at Test 2) to the cut-off point 6 of the original scoring would result in a cut-off of 1.83–1.78 (Test 1, Test 2), which is indeed closer to 2 than 3.

## Conclusions

If the simplified scoring (0 to 8) of the Actionable questionnaire is used, in MS patients the cut-off point 2 is likely to be more accurate than the cut-off point 3, especially by preventing a considerable number of patients from being withheld referral to an urologist (false negatives). Further, our data suggest that preferentially the Actionable item scoring should be as described originally by Bates et al., from 0 to 3, which results in a total score ranging from 0 to 24 [1]. Future studies of the Actionable in MS patients are needed to prospectively assess the sensitivity and specificity of the cut-off points 3 and 2 with respect to clinician-based assessments of whether or not urological treatment is needed.

## Abbreviations

ABSST: Actionable bladder symptom screening tool–16 item version
FN: False negatives
FP: False positives
MS: Multiple sclerosis
NPV: Negative predictive value
PPV: Positive predictive value
RR: Relapsing remitting
SD: Standard deviation
TN: True negatives
TP: True positives
VPN: Virtual private network

## References

[1] Bates D, Burks J, Globe D, Signori M, Hudgens S, Denys P, Macdiarmid S, Nitti V, Odderson I, Ross AP (2013). Development of a short form and scoring algorithm from the validated actionable bladder symptom screening tool. BMC Neurol.

[2] Burks J, Chancellor M, Bates D, Denys P, Macdiarmid S, Nitti V, Globe D, Signori M, Hudgens S, Odderson I (2013). Development and validation of the actionable bladder symptom screening tool for multiple sclerosis patients. Int J MS Care.

[3] Cardozo L, Staskin D, Currie B, Wiklund I, Globe D, Signori M, Dmochowski R, MacDiarmid S, Nitti VW, Noblett K (2014). Validation of a bladder symptom screening tool in women with incontinence due to overactive bladder. Int Urogynecol J.

[4] Sport MoHWa: Dutch Medical Research Involving Human Subjects Act (WMO). International Publication Series Health, Welfare and Sport. 1997;(2):1–34.

[5] Lechner-Scott J, Kappos L, Hofman M, Polman CH, Ronner H, Montalban X, Tintore M, Frontoni M, Buttinelli C, Amato MP (2003). Can the expanded disability status scale be assessed by telephone?. Mult Scler.

[6] Eusebi P (2013). Diagnostic accuracy measures. Cerebrovasc Dis.

[7] Langdon DW (2011). Cognition in multiple sclerosis. Curr Opin Neurol.

[8] Gold SM, Schulz H, Monch A, Schulz KH, Heesen C (2003). Cognitive impairment in multiple sclerosis does not affect reliability and validity of self-report health measures. Mult Scler.

